# nc886, a non-coding RNA of anti-proliferative role, is suppressed by CpG DNA methylation in human gastric cancer

**DOI:** 10.18632/oncotarget.2047

**Published:** 2014-05-31

**Authors:** Kwang-Soo Lee, Jong-Lyul Park, Kwanbok Lee, Lauren E. Richardson, Betty H. Johnson, Hyun-Sung Lee, Ju-Seog Lee, Sang-Bae Kim, Oh-Hyung Kwon, Kyu Sang Song, Yong Sung Kim, Hassan Ashktorab, Duane T. Smoot, Sung Ho Jeon, Seon-Young Kim, Yong Sun Lee

**Affiliations:** ^1^ Department of Biochemistry and Molecular Biology, The University of Texas Medical Branch, Galveston, TX, USA; ^2^ Department of Life Science and Center for Aging and Health Care, Hallym University, Chuncheon, Korea; ^3^ Medical Genomics Research Center, KRIBB, Daejeon, Korea; ^4^ Department of Functional Genomics, University of Science and Technology, Daejeon, Korea; ^5^ Lamar University, Beaumont, TX77710, USA; ^6^ Department of Systems Biology, The University of Texas MD Anderson Cancer Center, Houston, TX, USA; ^7^ Department of Pathology, College of Medicine, Chungnam National University, Daejeon, Korea; ^8^ Departments of Medicine, Howard University, Washington, D. C., USA; ^*^ co-first authors

**Keywords:** nc886, gastric cancer, CpG DNA methylation, cell proliferation, tumor suppressor

## Abstract

nc886 is a 101 nucleotide long non-coding RNA that has been designated as a precursor microRNA or a vault RNA based upon it sequence. nc886 has also been suggested to be a tumor suppressor, mainly inferred by its expression pattern as well as its genomic location at human chromosome 5q31, a locus for a tumor suppressor gene(s). However, legitimate data based on nc886's correct identity for its functional cellular roles as a tumor suppressor have not been provided yet. Here we have investigated nc886 in gastric cancer where its expression is suppressed due to CpG DNA hypermethylation at its promoter region in a cohort of paired tumor/normal tissues from 88 gastric cancer patients. CpG hypermethylation of nc886 and thus its diminished expression is significantly associated with poor survival in these cancer patients. nc886 inhibits cell proliferation when ectopically expressed in gastric cancer cells. nc886's tumor suppressive role is corroborated by the induction of well-known oncogenes such as FOS, NF-κB, and MYC upon its knockdown. All these activities of nc886 are undoubtedly independent of mature microRNA or vault RNA. Our data indicate that nc886 is a putative tumor suppressor and could potentially be used as a diagnostic marker in gastric cancer.

## INTRODUCTION

Gastric cancer is a frequently occurring, lethal cancer. In 2008 according to worldwide estimation 989,600 new cases were diagnosed and 738,000 patients died which ranked gastric cancer fourth in incident rate and the second in mortality among all cancers [1]. Failure of early diagnosis accounts for the high mortality rate. Most gastric cancers can be cured by surgical resection at early stages; however, patients in advanced stages have poor prognosis. Therefore, the development of early diagnostic molecular marker(s) is clinically important in lessening this high mortality rate.

We have recently identified nc886, a 101 nucleotide (nt) long non-coding RNA (ncRNA) that is abundantly present in the cytoplasm of human cells [2]. Although nc886 has been known as a microRNA (miRNA) precursor or a vault RNA (hence its aliases are pre-miR-886 or vtRNA2-1), our data show that nc886 is neither implicated in the miRNA pathway nor present in the vault complex [2]. nc886 is encoded on human chromosome 5q31, which is sometimes deleted in leukemia and thus is considered to harbor a tumor suppressor gene(s) [3, 4]. Consistently, the expression of nc886 is suppressed in a number of cancer cells relative to non-malignant cells [2, 5, 6]. This expression pattern is intriguing given that nc886 is transcribed by RNA polymerase III (Pol III) [6-8] whose activity is generally considered to be elevated in malignancies [reviewed in [9].

Until now, nc886's best characterized role is to bind PKR (*P*rotein *K*inase *R*NA-activated) and inhibit its activity [2, 5, 10]. Knockdown of nc886 provokes the PKR pathway which is usually pro-apoptotic through the phosphorylation of eIF2α and the resultant inhibition of global cellular protein synthesis. This anti-apoptotic role for nc886 is discordant with what would be anticipated for a tumor suppressor, whereas its expression pattern is concordant. To reconcile this contradiction, we have proposed a tumor surveillance model [5, 11]. In this model, the cell death upon nc886 suppression serves as a checkpoint to eliminate nascently transformed cells before manifestation into clinically detectable malignancies. Malignant cells lacking nc886 are the ones that have survived this checkpoint; for example, by the overexpression of eIF2B which neutralizes the pro-apoptotic effect of phospho-eIF2α. Our tumor surveillance model involves a tumor-sensing role for nc886; however, separate evidence is needed to prove its tumor suppressor role.

One of the most common epigenetic modifications in the mammalian genomic DNA is its methylation at cytosine residues in CpG dinucleotides. Tumor suppressor genes, mostly protein-coding genes and thus transcribed by RNA polymerase II, are frequently silenced during tumorigenesis by CpG methylation at their promoter region [reviewed in [12]. Although CpG methylation in Pol III transcription has been addressed in a few reports [13-15], its in-depth mechanism or biological significance is still unknown.

In this paper, we have surveyed nc886 expression and its CpG DNA methylation in a cohort of gastric cancer patients and investigated its methylation and expression further in gastric cell lines. Also, we have provided evidence supporting nc886's tumor suppressor role and clarified that identity once more. These data concerning the regulation and function of nc886 support its utilization as an early diagnostic molecular marker in gastric cancer.

## RESULTS

### nc886 expression is suppressed in gastric cancer tissues

**Figure 1:**
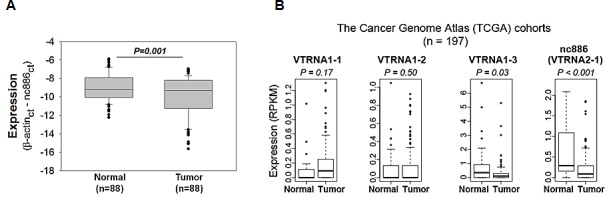
nc886 expression in clinical specimens from gastric cancer patients and TCGA cohorts A. nc886 expression levels, as expressed in Ct (cycle threshold) values relative to β-actin (“β-actin_ct_ - nc886_ct_” on y-axis), in tumor samples and their adjacent normal tissues from 88 patients. B. Expression of nc886 and three vault RNA genes in gastric cancer from the TCGA cohort (n = 197). Expression (RPKM) in y-axis denotes Reads Per Kilobase per Million reads.

To profile nc886 in gastric cancer, we measured its RNA expression in pairs of normal and tumor tissues from 88 patients. Our real-time RT-PCR data (Fig 1A) indicated that the expression level of nc886 was lower in a subpopulation of tumor tissues (mean ± SD: -9.93 ± 2.19) than in normal tissues (mean ± SD: -9.03 ± 1.47). Although the fold-difference was modest, the decrease was statistically significant (P=0.001) and corroborated by our other data that will be shown later.

We next examined nc886 expression in an independent cohort of gastric cancer from The Cancer Genome Atlas (TCGA) project. Coincided with our data, nc886 expression was significantly decreased in tumor tissues (P<0.001, Fig 1B) while the expression of three vault genes was either increased or not decreased as much as nc886. It is worthy to note that the vast majority of gastric cancer patients in the TCGA cohort were ethnically white while our patients were all Asians, suggesting that suppression of nc886 in gastric cancer is universal across different ethnic backgrounds.

### Suppressed expression of nc886 is due to its DNA hypermethylation in gastric cancer cell lines

**Figure 2:**
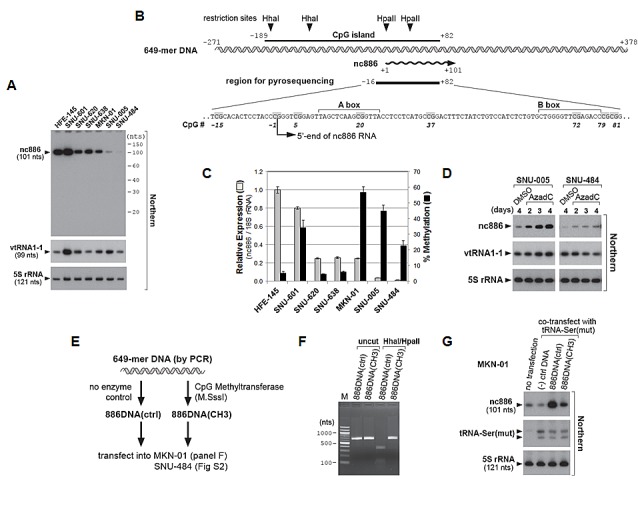
nc886 expression suppression in gastric cancer cell lines through CpG DNA methylation A. Northern hybridization of nc886, vtRNA1-1, and 5S rRNA (control for equal loading) in indicated gastric cell lines. Molecular sizes from 5′-^32^P-labeled Decade markers (10 nt ladder) are indicated on the right. B. Diagram depicting a genomic region encoding nc886. A 649 nt DNA segment (also used in panel E-G) is illustrated in a double helix and nc886 RNA in a wavy line with an arrowhead indicating the direction of transcription. All nt coordinates are numbered referring to the 5'-end nt of nc886 RNA as +1. In the region for pyrosequencing, CpG dinucleotides are highlighted in grey and numbered consistently with Fig S3. A and B boxes (Pol III recognition elements) are also indicated. C. nc886 RNA expression and CpG DNA methylation levels. nc886RNA expression was measured by qRT-PCR. Ct values of each gene were converted to relative quantity (2^-Ct^) and normalized to 2^-Ct^ values from 18S rRNA. The value of HFE-145 was set as 1 (grey bars on the left y-axis). Percent methylation of cytosine at seven CpG sites (shown in Fig 2B, except for CpG #37) was measured by pyrosequencing. The seven values (shown in Fig S3) were averaged and plotted (black bars on the right y-axis). For both measurements, an average and the standard deviation were calculated from triplicate samples. D. Northern hybridization after treatment with 10 μM of AzadC. All other descriptions are the same as in panel A. E-G. Transfection of an *in vitro* methylated nc886 DNA fragment. The experimental scheme is illustrated in panel E. After M.SssI enzyme treatment (or no enzyme control), methylation of the 649-mer DNA (see panel B and E) was assured by digestion with methylation-sensitive restriction endonucleases *Hha*I/*Hpa*II (panel F). “M” (lane 1) indicates molecular size markers whose sizes in nts are indicated on the left. “(-) ctrl DNA” (in panel G) denotes a 597-mer DNA fragment from an irrelevant gene, MKRN1. At 24 hrs after transfection of indicated DNA into MKN-01 cells (lane 2-4), nc886 and 5S rRNA were measured by Northern hybridization (panel G). Comparable transfection efficiencies were confirmed by a tRNA-Ser(mut) signal from the co-transfected plasmid “pCR-tRF1001_338(mut1)”. This signal was almost absent in the untransfected sample (lane 1).

We measured nc886 expression also in gastric cell lines (Fig 2A). HFE-145 is a gastric epithelial cell line immortalized by SV40 large T antigen and telomerase [16]. HFE-145, as a representative of immortal but non-malignant cells, expressed nc886 abundantly. In contrast, nc886 expression was decreased in most gastric cancer cell lines, except SNU-601. Unlike nc886, vtRNA1-1, a canonical vault RNA, was constitutively expressed in all the cell lines. This pattern was similar to what we had observed in other sets of cell lines from lung cancer, head-neck cancer, and cholangiocarcinoma [2, 5]. It should be noted that a single band at ~100 nts but no other band was seen in our Northern hybridization (Fig 2A and S1).

Our *in silico* analysis [using http://cpgislands.usc.edu/, [17] detected a CpG island at nt coordinates from -189 to +82 (Fig 2B), with +1 position being the 5'-end of nc886 RNA [2]. This is a strong CpG island with Obs_CpG_/Exp_CpG_ (an observed to expected CpG ratio) of 0.90 and a (C+G) ratio of 62.7%, which are far above threshold values [0.60 and 50% respectively, [18]. To investigate the role of this island in nc886, we measured its methylation status by bisulfite sequencing (Fig S2). The CpG island was hypermethylated (31.0-48.0%) in two cell lines barely expressing nc886 (SNU-005 and SNU-484), but was hypomethylated (0.5-7.0%) in three cell lines expressing nc886 (HFE-145, SNU-620, and SNU-638). SNU-601 was again aberrant with high nc886 expression as well as CpG hypermethylation. We selected seven CpG dinucleotides within the nc886 CpG island region, performed pyrosequencing, and found that nc886 CpG island methylation was negatively correlated with its expression (Fig 2B-C and S3).

Most likely, CpG hypermethylation might be a mechanism to suppress nc886 RNA expression. To prove this, we treated cells with AzadC, a DNA methyltransferase inhibitor. We used SNU-005, SNU-484, and MKN-01 cells, all of which exhibited CpG hypermethylation and low nc886 expression (Fig 2C). AzadC treatment resulted in elevated nc886 expression (SNU-005 and SNU-484, Fig 2D; MKN-01, data not shown). Although this result was in agreement with our anticipation, an indirect effect of AzadC, for example by modulating a transcription factor for nc886 expression, could not be ruled out.

To provide direct evidence, we compared CpG-methylated versus -unmethylated DNA for nc886 expression (Fig 2E). nc886 is transcribed by Pol III and possesses promoter elements (A and B Box in Fig 2B) within the transcript. We found that a 649 nts genomic DNA fragment (as shown in Fig 2B) was sufficient to express nc886 when transfected into most human cell lines. The 649 nt DNA was *in vitro* methylated and the methylation was validated by digestion with methyl-sensitive restriction enzymes *Hha*I/*Hpa*II (Fig 2F). When transfected into MKN-01 and SNU-484 cells, nc886 was expressed efficiently from the un-methylated 649 nt DNA fragment, but barely from the methylated one (“886DNA (ctrl)” versus “886DNA (CH3)” in Fig 2G and Fig S4). All our data consistently indicated that CpG hypermethylation at the nc886 promoter region is one reason for nc886 suppression in gastric cancer.

### Hypermethylation of nc886's CpG island in gastric tumor tissues relative to adjacent non-tumor tissues

**Figure 3:**
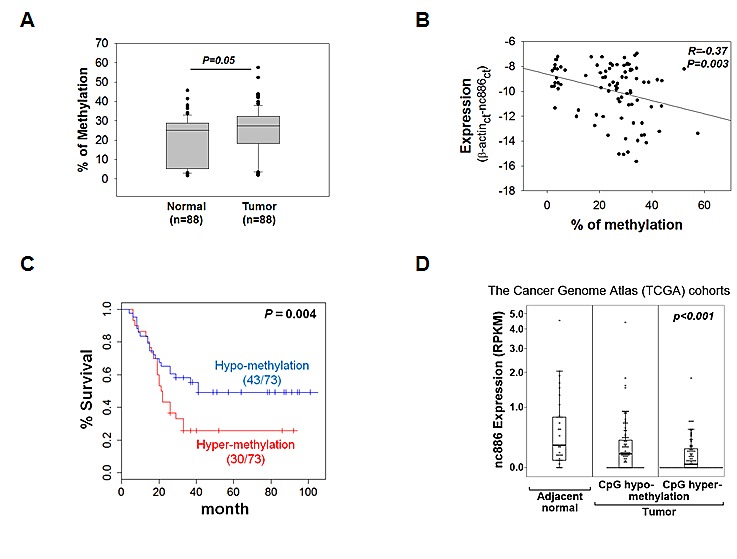
nc886 CpG methylation in the clinical specimens and the TCGA cohorts A. nc886 CpG methylation levels (averaged from four CpG sites; #-15, −1, 5, and 20) in the 88 patient samples. B. Correlation between nc886 CpG methylation (panel A) and RNA expression (Fig 1A) from the patient data. C. Kaplan-Meier curves between low (< 30.15%) and high (>=30.15%) nc886 CpG methylation groups. 15 samples were excluded in survival analysis due to no survival data. D. A box plot showing negative correlation between nc886 RNA expression and CpG DNA methylation in gastric cancer (n=155) from the TCGA cohort. 25 normal adjacent tissues were analyzed together.

To assess CpG methylation in the 88 patient samples, we performed pyrosequencing experiments for four CpG sites (CpG #-15, −1, 5, and 20 in Fig 2B) and found it to be significantly (P=0.05) elevated in tumors (Fig 3A). Methylation levels in a tumor and its adjacent normal tissue were 24.60 ± 12.70 and 21.09 ± 11.72 (mean ± SD), respectively. In 58 of the 88 cases, CpG methylation in a tumor tissue was higher than its paired adjacent normal tissue. Comparison of the methylation data to the expression data indicated a negative-correlation between the two (R= −0.37, P=0.003) (Fig 3B). Most importantly, Kaplan-Meier curves revealed significant (P=0.004) shorter survival of patients with nc886 hypermethylation in tumors (Fig 3C), suggesting that nc886 CpG methylation can be a prognostic marker in gastric cancer. Association between nc886 methylation and clinical pathological characteristics was summarized in Table S1. The negative correlation between expression and methylation was also observed in the independent TCGA cohort, where suppression of nc886 expression was more pronounced in tumors with CpG hypermethylation (P<0.001, Fig 3D).

### nc886 is anti-proliferative in gastric cancer cells with low endogenous nc886 expression

**Figure 4:**
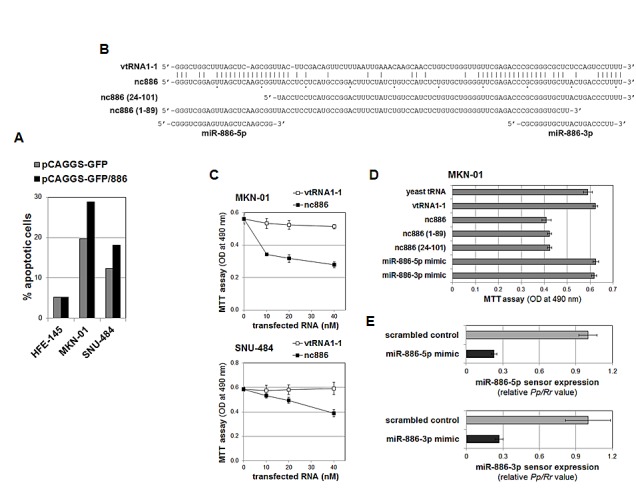
nc886 inhibition of cell proliferation independently of mature miRNAs A. The percentage of apoptotic cells (Annexin V-positive) in transfected cells (GFP-positive), measured by FACS analysis (see also Fig S6 for raw FACS data). After transfection of pCAGGS-GFP or pCAGGS-GFP/886, HFE-145 cells were grown in the presence of 2.5 mg/ml of G418 for three weeks to yield HFE-control and HFE-nc886 cells respectively (see Fig S5 for their nc886 expression levels). In contrast, SNU-484 and MKN-01 cells were grown for five days without G418 and harvested for FACS. B. The sequence alignment of nc886 to vtRNA1-1, the deletion mutants, and mature miRNAs. Identical nts between nc886 and vtRNA1-1 are indicated by vertical lines. C. Cell proliferation (MTT) assays at 24 hrs (for MKN-01) or 72 hrs (for SNU-484) after transfection of *in vitro* transcribed nc886 or vtRNA1-1 at indicated concentrations (x-axis). At each titration point, the total amount of transfected RNA was adjusted to 120 ng (per 96-well) by yeast tRNA. An average and a standard deviation from triplicate samples are shown. D. Cell proliferation assays at 24 hrs after transfection of indicated RNAs at 10 nM. All other descriptions are the same as in panel C. E. Dual luciferase assays at 24 hrs after transfection of indicated miRNA mimics (at 10 nM) and sensor plasmids. A firefly luciferase value (*Pp*) from the sensor plasmid (with a perfect complementary target site of miR-886-5p or -3p at the 3'-untranslated region of *Pp* open reading frame) was normalized to the renilla value (*Rr*) from the co-transfected pRL-SV40. The *Pp/Rr* value was again normalized to the *Pp/Rr* value from pcDNA3.1-Zeo(+)-Pp (a control firefly luciferase plasmid without a miRNA target sequence), yielding a relative *Pp/Rr* value (y-axis). The values from “control scrambled oligo” were set as 1. An average and a standard deviation from triplicate samples are shown.

nc886's expression pattern suggested that it is a tumor suppressor. To assess such a role, we sought to generate transgenic gastric cell lines stably expressing nc886. Despite several attempts in gastric cancer cells with low endogenous nc886 expression (such as SNU-484 and MKN-01, see Fig 2A), we failed to recover cells expressing nc886. This failure was not due to technical problems, because we obtained cells stably expressing nc886 (“HFE-nc886” versus “HFE-control” in Fig S5) from the HFE-145 cell line that expressed a high level of endogenous nc886 (Fig 2A). Compared to HFE-control cells, HFE-nc886 cells grew well and exhibited a similar degree of apoptosis as measured by Annexin V staining (Fig 4A and S6). In contrast, more apoptotic cells were seen after transient transfection of nc886 expressing plasmid into SNU-484 and MKN-01 (Fig 4A and S6, indicating that re-expression of nc886 into these cells was deadly.

One drawback to the above experiments employing plasmid DNA was the uptake of DNA by only a minor population of cells. To examine nc886's effect on cell proliferation more clearly, we transfected *in vitro* transcribed nc886, because nc886 RNA is only 101 nts long (Fig 4B) and can be transfected much more efficiently. In agreement with the above results, nc886 inhibited proliferation of MKN-01 and SNU-484 cells, but a canonical vault RNA vtRNA1-1 did not (Fig 4B and C).

A few recent papers have reported growth suppressive functions of mature miR-886-3p [19-21]. However, we consistently failed to detect mature miRNAs [Fig 2A and S1 [2, 5]. One possibility, though very unlikely, is that nc886 was processed to mature miRNAs at a sub-detectable level and this minute quantity of mature miRNAs was functional. To exclude this possibility, synthetic mature miRNA mimics were transfected at the same molar concentration of the synthetic nc886. The miRNA mimics have no effect on cell proliferation (Fig 4B and D), even though they were active as a mature miRNA in the transfected cells as shown by the suppression of luciferase expression from co-transfected sensor plasmids harboring a miRNA target site (Fig 4E). Furthermore, deletion of the 5'- or 3'-end of nc886 did not impair its capability to inhibit cell proliferation (Fig 4B and D). Therefore, it is clear that nc886's anti-proliferative activity resided in its central portion and was independent of mature miRNAs.

### A gene expression signature upon nc886 knockdown supports its tumor suppressive role

**Figure 5:**
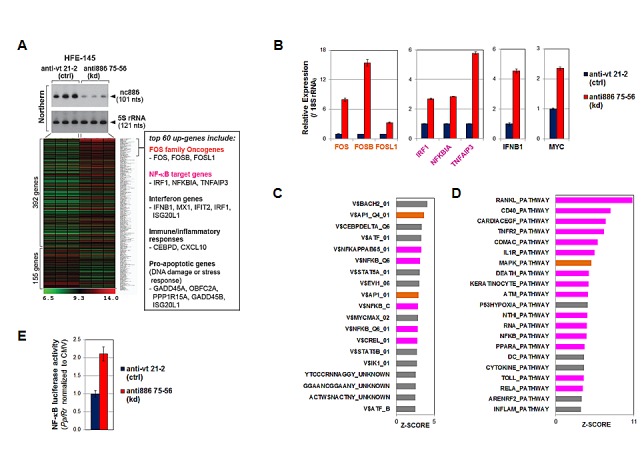
nc886 knockdown activation of oncogenic FOS, NF-κB, and MYC as well as other pathways A. A heatmap of regulated genes upon nc886 knockdown (kd) versus negative control (ctrl) by anti-oligo “anti886 75-56” and “anti-vt 21-2” respectively. Cells were harvested for RNA preparation at 24 hrs after transfecting 88 nM of each oligo by a reverse transfection protocol (cells plated after mixing with a transfection mixture). Knockdown of nc886 in triplicate transfections was shown by Northern hybridization. The top 60 up-regulated genes were individually scrutinized in Entrez Gene and PubMed, and those representing our pathway analysis (in panel C and D) were chosen and summarized in the box on the right. B. qRT-PCR measurement of indicated genes. The Ct values (from triplicate samples) were processed as described in Fig 2C. Control knockdown (“anti-vt 21-2”) values were set as 1 (y-axis). C. TFT (transcription factor targets) analysis. The most enriched TFTs (Z-SCORE cutoff = 2.5) upon nc886 knockdown are shown; orange bars, AP-1; magenta bars, NF-κB. D. BioCarta pathway analysis (Z-SCORE cutoff = 3.5). All other descriptions are the same as panel C. E. NF-κB promoter luciferase assays at 24 hrs after transfection of anti-oligos, as described in panel A. The data processing and normalization was as described in Fig 4E.

Next, we evaluated nc886's tumor suppressive loss-of-function role. To understand intracellular events triggered by nc886 suppression, we examined global gene expression profiles by mRNA array after transfecting a synthetic anti-oligo into non-malignant HFE-145 cells. It is worth noting that the anti-oligo (“anti886 75-56”) targets nts 56-75 of nc886 and thus should have no direct effect on mature miRNAs even if any mature miRNA were produced (see Fig 4B). We used an anti-oligo targeting the canonical vault RNA (“anti-vt 21-2”) as a control. Efficient knockdown was confirmed by the disappearance of the 101 nt band in Northern hybridization (Fig 5A).

Upon nc886 knockdown, the expression of 392 and 155 genes were significantly increased and decreased respectively (cutoff = 1.5 fold, Fig 5A and Table S2). Among the top 60 up-regulated genes (excerpted from Table S2 and listed in a box in Fig 5A), the first noticeable ones were the three FOS family proteins (FOS, FOSB, and FOSL1). Their induction upon nc886 knockdown was confirmed by qRT-PCR (Fig 5B). The FOS transcription factor is a subunit of the AP-1 transcription factor and well-known oncogene of a transforming potential when aberrantly expressed [22]. Thus the induction of FOS upon nc886 knockdown could be one way that the suppression of nc886 promotes gastric carcinogenesis. Our analysis from the whole array data also indicated that genes with an AP-1 target site(s) tended to be elevated (orange bars in Fig 5C) and genes in the MAP kinase pathway also were enriched (orange bar in Fig 5D; also see Fig S7A for the gene network).

nc886 knockdown also led to an increased regulation of other oncogenic pathways. Among them, NF-κB activation was the most pronounced, as shown by the induction of *bonafide* NF-κB target genes (Fig 5A and B) and the enrichment of genes harboring a NF-κB target site(s) at their promoters (magenta bars in Fig 5C). We also experimental confirmed the elevation of NF-κB promoter activity by luciferase report assays (Fig 5E). Most pathways in Fig 5D (magenta bars) involved NF-κB and many of the induced genes were connected to the NF-κB network (Fig S7B). MYC oncogene was also significantly up-regulated upon nc886 knockdown, as shown by our qRT-PCR measurement (Fig 5B) and pathway analysis (V$MYCMAX_02 in Fig 5C and Fig S7C).

**Figure 6:**
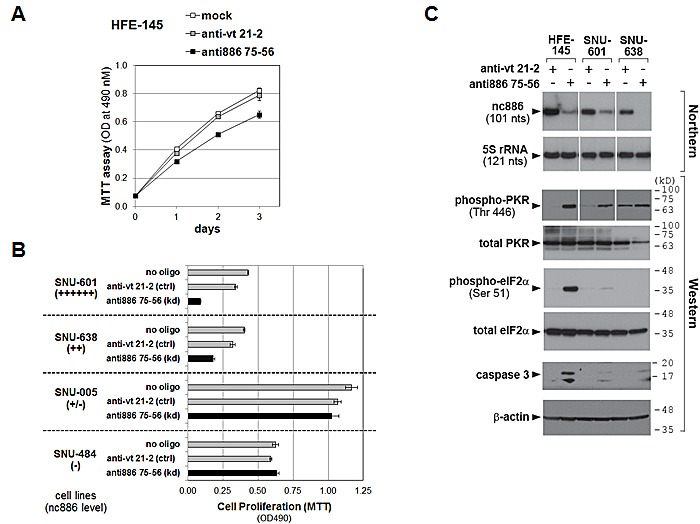
Cell proliferation and the PKR pathway upon nc886 knockdown in gastric cells A-B. Cell proliferation (MTT) assays after mock transfection (no oligo included during transfection) or transfection of each anti-oligo (at 100 nM) as described in Fig 5A. MTT was performed at day 1 (SNU-601) or day 2 (all the other cell lines) after transfection C. Northern hybridization and Western blot of indicated RNA and proteins after transfection of indicated anti-oligos. Molecular size markers in kilodalton (kD) are shown on the right for the Western blot. HFE-145 and SNU-638 cells were harvested at 24 hrs after reverse-transfection. SNU-601 cells were transfected twice at 0 and 24 hrs by a forward transfection protocol (a transfection mixture added to pre-plated cells). Cells were harvested at 24 hrs after the second transfection.

In addition to the activation of oncogenic pathways, we detected the induction of genes in interferon, innate immune response, and inflammation pathways (Fig 5A-D). This was not surprising, since nc886 is a PKR inhibitor and its suppression indeed activated PKR (shown later in Fig 6C). Together with these genes, several other pro-apoptotic genes were also induced (Fig 5A). Presumably, nc886 knockdown followed by PKR activation would be identified by cells as a viral infection, committing “infected” cells to death.

In the gene expression profile upon nc886 knockdown, we failed to see enrichment of predicted target genes of mature miR-886-3p and -5p; this failure is opposed to the anticipated result if nc886 were a genuine miRNA precursor (Fig S8). Thus, our gene expression analysis also supported our evaluation that nc886 is not a miRNA precursor in gastric cancer.

### Acute cellular response to nc886 knockdown of nc886 is cell death; one mechanism for this is the classical PKR pathway

nc886 knockdown inhibited proliferation of the non-malignant HFE-145 cell line (P<0.001, Fig 6A) as well as two gastric cancer cell lines expressing nc886 (SNU-601 and SNU-638, Fig 6B). This should not be a non-specific toxic effect of the anti-oligo, because its transfection did not affect proliferation of nc886-deficient cell lines (SNU-005 and SNU-484, Fig 6B).

If nc886 is a *bonafide* tumor suppressor, a malignant phenotype would be expected especially in HFE-145 cells. However, our proliferation data were not so unforeseen, because nc886 is a repressor of PKR, which is usually regarded to be apoptotic when de-repressed. Indeed, nc886 knockdown provoked the canonical PKR-eIF2α cell death pathway in HFE-145 cells, as shown by activation of PKR (indicated by phospho-PKR in Fig 6C) and the consequent eIF2α phosphorylation that led to apoptosis (Fig 6C). In contrast to the non-malignant HFE-145 cells, phospho-eIF2α and caspase-3 cleavage were barely seen in SNU-601 and SNU-638 cells, indicating that the PKR-eIF2α cell death pathway did not operate normally in these two gastric cancer cell lines. Intriguingly, malfunction of the PKR-eIF2α pathway in gastric cancer cell lines but its normal operation in non-malignant HFE-145 cells is what we observed previously in cholangiocarcinoma cell lines and non-malignant cholangiocyte respectively [5]. Nonetheless, nc886 knockdown inhibited the growth of SNU-601 and SNU-638 cells (Fig 6B) and this might be attributed to other death pathways. For example, the pro-apoptotic genes seen in our array data might be the cause (Fig 5A). The cell-type dependent mechanism for nc868's effect on cell growth remains to be clarified.

## DISCUSSION

This is the first report on nc886 in gastric cancer patients; our initial identification and subsequent studies have been conducted mostly in cultured cell lines. Only two studies examined nc886 in a large cohort of clinical samples [6, 20]. Although they measured CpG DNA methylation of nc886, these studies lacked RNA expression data or prudent verification of its identity. Here, we have obtained a complete set of DNA methylation and RNA expression data from 88 patients which render our finding clinically significant. We have also provided evidence for nc886's tumor suppressive role by our experiments which were designed based on its correct identity.

Although our initial paper proved that nc886 is neither a miRNA precursor nor a vtRNA [2], we interrogated nc886's identity once more. nc886 is undoubtedly a 101 nt sized RNA, as indicated by a clean band in Northern hybridization (shown in Fig 2A and S1) and RT-PCR amplification using two primers at both ends (Fig 1A and 2C). nc886 is functionally distinguished from mature miRNAs or vtRNA1-1, as consistently indicated by several experiments (Fig 4-6). All our data here coincide with our previous papers [2, 5, 10] and ascertain that nc886 is a distinct ncRNA.

Recently, a growing number of papers have been reporting aberrant expression of miR-886-5p and -3p in various malignancies [6, 19-21, 23-27], mostly because nc886 was once classified in a precursor miRNA (namely pre-miR-886) and therefore included in probe sets in several miRNA array platforms. We strongly presume that nc886, rather than the mature miRNAs, is what most arrays detected, because Northern data from our laboratory and others unequivocally demonstrated that the mature miRNAs are barely produced [2, 5, 6, 10, 28]. In many hybridization-based arrays, nc886 is thought to be what the two probes (-886-5p and -3p) detected, similarly to usual mRNA arrays where a single transcript is detected by multiple probes. This should be especially true, when miR-886-5p and -3p had similar array values and tendency among samples [2, 21, 23-25, 29-31]. In the case of miRNA arrays employing stem-loop PCR such as Taqman assays [32], nc886 and miR-886-3p are not distinguished in principle, because they share the same 3'-end. This explains why miR-886-3p was more prevalently reported than miR-886-5p. Given that nc886 is very abundant (10^5^ copies per single HeLa cell [2]), its degradation intermediates would also be captured as if they were the mature miRNAs.

Suppression of nc886 expression in gastric cancer suggested its tumor suppressor role. This was congruent with its anti-proliferative activity (Fig 4) and was corroborated by provocation of several oncogenic pathways upon nc886 knockdown (Fig 5). Yet, nc886 suppression alone was not sufficient to drive cells into malignancy, like most tumor suppressor genes. Sudden depletion of nc886 and the resultant PKR activation are likely to mimic a situation similar to viral assault and provoke the PKR-eIF2α apoptotic pathway especially in non-malignant cells. It should be noted that nc886 knockdown induced several other pro-apoptotic genes, for example in the DNA damage response (Fig 5A), in addition to the PKR-eIF2α apoptotic pathway. The effect of nc886 depletion, which appears to be pleiotropic and dependent on cell types, should be delineated before determining its precise role during tumor etiology/progression.

Although studies on nc886 are still at a primitive stage, we expect that nc886 will soon emerge as an important molecule in cancer. There are many outstanding questions to be answered: for example, what is the very first signal that triggers nc886 methylation? How does cytoplasmic nc886 cause altered gene expression? Until now, nc886's direct molecular link is only PKR, which could explain the nc886-NF-κB relationship; but we speculate that nc886 interacts also with other molecules which remain to be identified.

Our findings here provide a future possibility to utilize nc886 for gastric cancer diagnosis and prognosis, especially by measuring its CpG DNA methylation. nc886's DNA methylation is a better marker than its RNA expression for the following reasons. First, DNA is more stable than RNA. And second, nc886 DNA methylation is increased in cancer and thus its measurement is tolerant to some contamination of surrounding non-malignant tissues. Although nc886 is certainly regulated by CpG methylation, other factors such as transcription factors are also likely to be involved, as suggested by the case of SNU-601 in which both nc886 RNA expression and CpG methylation were high. Another critical question is whether CpG hypermethylation occurs early in gastric tumorigenesis or not. Thus, its clinical application must be preceded by a better understanding of nc886 regulation and the collection of more patient data including prospective cohorts.

## MATERIAL AND METHODS

### Cell lines and tissue samples

Sources for gastric cell lines and their culture conditions are described in Supplemental Information. All gastric cell lines were authenticated by STR (Short Tandem Repeats) genotyping at the Korean Cell Line Bank (data available upon request). 88 pairs of a tumor and its adjacent normal gastric tissue sample were obtained from Chungnam National University Hospital (Korea) with the informed consent and approval of the Internal Review Board at Chungnam National University.

### Pyrosequencing of the nc886 promoter region

Genomic DNA was isolated by a PureLink™ Genomic DNA kit (Invitrogen, Carlsbad, CA) and 1 μg was subjected to bisulfite-conversion using an EZ DNA methylation kit (Zymo Research, Orange, CA). Primers specific to A box in nc886 for pyrosequencing were as previously described [6]. Sequence information for pyrosequencing primers are in Table S3.

### Measurement and analysis of RNA

Total RNA from gastric cell lines was isolated by Trizol reagent (Invitrogen) and Northern hybridization was performed as described in [2]. Detailed procedures for qRT-PCR are described in Supplemental Information. The sequences of Northern probes and qRT-PCR primers are summarized in Table S3.

### Statistical analysis

In analyzing nc886 RNA expression and CpG methylation data from the 88 patients, we applied the Student's t-test to evaluate the significance in difference between tumors and adjacent normal tissues. To estimate nc886's significance (between high- versus low-CpG methylation groups) in patient survival, we applied Kaplan-Meier survival analysis and the log-rank test using the R software (version 2.6.1). Results with a p-value of < 0.05 were considered significant.

### Antibodies and other reagents

The source of antibodies has been described [2, 5]. 5-Aza-2'deoxycytidine (AzadC) was purchased from Sigma-Aldrich (St. Louis, MO), *Hha*I, *Hpa*II, and M.SssI were from New England Biolabs (Ipswich, MA). The Decade marker for small RNA Northerns was from Applied Biosystems/Ambion (Carlsbad, CA); DNA and protein size markers were from GenDepot (Barker, TX).

### Plasmid DNAs, *in vitro* methylated DNAs, and RNAs

Plasmid “pCR-tRF1001_338(mut1)” expressing a mutant tRNA was constructed as described in Supplemental Information and was used for transfection efficiency control in Fig 2G. A plasmid “pCAGGS-GFP” was constructed by inserting GFP open reading frame into *Xho*I/*Bg*lII sites of the pCAGGS-neo vector and used for a control plasmid for transfection and FACS experiments. The nc886 DNA fragment (“649-mer DNA” depicted in Fig 2B) and a negative control DNA fragment (597-mer DNA from an irrelevant MKRN1 gene) were PCR-amplified on genomic DNA and its sequence was confirmed to be identical to the reference human genome sequence (http://genome.ucsc.edu/). The 649-mer nc886 DNA fragment was cloned into *Sal*I/*Spe*I sites of pCAGGS-GFP to generate a plasmid “pCAGGS-GFP/886”. Both PCR fragments (nc886 and MKRN1) were *in vitro* methylated by M.SssI per manufacturer's instruction. Synthetic anti-sense oligonucleotides (anti-oligos) against nc886 and vtRNA1-1), *in vitro* transcribed RNA, and yeast tRNA were purchased or prepared as described previously [2, 5, 10]. DNA templates for *in vitro* transcription of vtRNA1-1, nc886, and its deletion mutants were PCR-amplified with primers whose sequences are summarized in Table S3. Mimic oligonucleotides for mature miR-886-5p and -3p was purchased from Applied Biosystems/Ambion.

### Transfection, luciferase assay, and cell proliferation assay

Anti-oligos were transfected with Lipofectamine™ RNAiMAX reagent (Invitrogen). Plasmid DNA, DNA fragments, *in vitro* transcribed RNA and miRNA mimics were transfected with Lipofectamine™ 2000 reagent (Invitrogen). Luciferase assays and cell proliferation assays were done as described previously [2].

### FACS and apoptosis assay

Apoptosis was measured by the Annexin V-PE Apoptosis Detection Kit (Abcam, Cambridge, MA). FACS was run in FACSCanto™ II (BD Biosciences, San Jose, CA) and data were analyzed by FACSDiva 7 software (BD Biosciences).

### Whole genome expression assay and gene set analysis

mRNA array was performed by using a TotalPrep™ RNA amplification kit and a HumanHT-12 v4.0 Expression BeadChip kit (Illumina, San Diego, CA) (see Supplemental Information for details). The array data were deposited in the Gene Expression Omnibus (accession number GSE51067).

In gene set and pathway analysis, Z-scores and p-values were calculated by using the PAGE (Parametric Analysis of Gene Set Enrichment) method [33] with MSigDB (ver 3.0) gene sets [34]. For network analysis, we utilized the Ingenuity Pathway Analysis package (IPA; http://www.ingenuity.com) using differentially expressed genes (cutoff = 1.5 fold) upon nc886 knockdown.

## SUPPLEMENTARY TABLES




